# Soluble Fiber and Insoluble Fiber Regulate Colonic Microbiota and Barrier Function in a Piglet Model

**DOI:** 10.1155/2019/7809171

**Published:** 2019-12-23

**Authors:** Tingting Chen, Daiwen Chen, Gang Tian, Ping Zheng, Xiangbing Mao, Jie Yu, Jun He, Zhiqing Huang, Yuheng Luo, Junqiu Luo, Bing Yu

**Affiliations:** ^1^Institute of Animal Nutrition, Sichuan Agricultural University, Chengdu 611130, Sichuan, China; ^2^Key Laboratory of Animal Disease-Resistance Nutrition of Ministry of Education, Key Laboratory of Animal Disease-Resistant Nutrition, No. 211 Huimin Rood, Wenjiang District, Chengdu, Sichuan 611130, China

## Abstract

The main purpose of the present study was to assess the effect of soluble and insoluble fiber on colonic bacteria and intestinal barrier function in a piglet model. A total of 24 piglets (25 ± 1 d old; 7.50 ± 0.31 kg) were randomly allotted to 4 treatments: basal diet (control, CON), 1% insoluble dietary fiber (IDF) diet, 1% soluble dietary fiber (SDF) diet, and 0.5% insoluble fiber + 0.5% soluble dietary fiber (MDF) diet. The trial lasted 28 days. SDF-fed piglets showed a higher (*P* < 0.05) bacterial *a*-diversity (observed_species, chao1, and ACE) and a higher relative abundance of Proteobacteria and Actinobacteria, *Solobacterium*, *Succinivibrio*, *Blautia,* and *Atopobium* in colonic digesta than CON, IDF, and MDF groups (*P* < 0.05). At the same time, Bacteroidetes, Euryarchaeota, *Phascolarctobacterium*, *Coprococcus_1,* and *Prevotella_1* were significantly increased in the IDF group when compared with CON, SDF, and MDF groups (*P* < 0.05). Furthermore, Bacteroidetes and Enterobacteriaceae, *Selenomonas*, *Phascolarctobacterium,* and *Alloprevotella*(*P* < 0.05) were significantly higher in the MDF group than those in the other three groups (*P* < 0.05). SDF diet increased the concentrations of short-chain fatty acid (SCFA) in colonic digesta (*P* < 0.05) when compared with the CON group and enhanced weight index of the colon (*P* < 0.05) than the CON and IDF groups. Furthermore, compared with the CON group, SDF, IDF, and MDF diets all upregulated the mRNA expressions of claudin-1 (*CLDN-1*) in colonic mucosa (*P* < 0.05), SDF and IDF diets upregulated the mRNA expressions of mucin 2 (*MUC2*) (*P* < 0.05), SDF diet increased mRNA expressions of zonula occludens 1 (*ZO-1*) and occludin (*OCLN*), while the IDF group enhanced the secretory immunoglobulin A (sIgA) concentrations (*P* < 0.05), respectively. IDF and MDF diets decreased expressions of *TNF-α*(*P* < 0.05). We concluded that the influence of soluble fiber on colonic microbiota was more extensive than that of insoluble fiber. Moreover, soluble fiber could more effectively improve colonic barrier function by upregulating gene expressions of the gut barrier.

## 1. Introduction

In western countries, colonic disorders are serious health issues [1]. Finding methods to maintain colon health in individuals are of current interest [2]. A healthy colon involves a symbiotic balance among the gut microbiota, the integrity of the intestinal barrier, and minimizing inappropriate inflammatory responses [3, 4]. The controversies exist between dietary fiber intake and colonic disorders [5–7]. Due to the fermentation in the hindgut, the effect of dietary fiber on hindgut health involved the mutual bacteria and the formation of short-chain fatty acid (SCFA) [8, 9].

Dietary fiber was reported to stimulate health-promoting bacteria (*Bifidobacterium, Lactobacillus, Prevotella,* etc.) and suppress pathogenic bacterial species (*Escherichia coli,* etc.) [8, 10]. Short-chain fructooligosaccharide intake increased the abundance of cecal *Akkermansia* and *Blautia* [11]. Moreover, fructan and cellulose led to a difference in UniFrac distances of fecal microbiota and *a*-diversity [12]. Different composition and physicochemical properties of fiber may lead to variations in gut microbiota and SCFA production [13, 14] Dietary fiber has been classified into soluble fiber and insoluble fiber according to the solubility [15] and the influence of two fibers on colonic microbial composition and metabolism, and colonic barrier still needs to be further investigated.

We hypothesized that soluble fiber and insoluble fiber caused different effects on colonic microbiota and regulated colonic barrier. Inulin is a soluble fiber made of fructans, which can be rapidly fermented in the colon. ARBOCEL (a crude fiber concentrate), an insoluble fiber with high water-holding capacity, is made of lignocellulose. These two fibers were selected as supplementary dietary fibers.

All piglets were fed diets for 28 days, and then microbial composition, the content of SCFAs, and the barrier function of the colon were determined. The results could potentially provide some new leads toward understanding the effects of soluble fiber and insoluble fiber on microbial groups and barrier functions in the colon of nonruminant animals and humans.

## 2. Materials and Methods

### 2.1. Animals and Experimental Design

All experimental procedures were approved by the Animal Welfare Committee of Sichuan Agricultural University and performed in accordance with the National Research Council's Guide for the Care and Use of Laboratory Animals.

Twenty-four 25-day-old piglets (Duroc × Landrace × Yorkshire) with body weight (BW) of 7.50 ± 0.31 kg were randomly allotted to four groups with 6 replicates (3 males and 3 females). The four dietary treatments included CON diet (basal diet, maize-soybean meal), and fiber groups including IDF diet (1% insoluble fiber diet), SDF diet (1% soluble fiber diet), and MDF diet (0.5% insoluble fiber + 0.5% soluble fiber diet).

### 2.2. Animal Feeding and Sample Collection

Experimental diets were formulated to meet or exceed the nutrient requirement recommended by NRC (2012). Ingredient composition of the basal diet is presented in Table 1. 1% maize in the basal diet was replaced by 1% ARBOCEL, 1% inulin, and 0.5% ARBOCEL +0.5% inulin, respectively, in the three fiber groups. ARBOCEL was provided by German J.RETTENMAIER & Söhne Group (Shanghai, China), and inulin was supplied by Ci Yuan Biotech Company Limited (Shanxi, China). The concentrations of inulin and ARBOCEL were 92.15% and 90%, respectively (provided by the manufacturer). The experiment was conducted at the Research Base of the Institute of Animal Nutrition of Sichuan Agricultural University. All pigs were housed individually in metabolism cages (1.5 m × 0.7 m × 1.0 m). The room lighting was natural with the temperature maintained at 26–28°C and relative humidity controlled at 60%–70%. Piglets were fed 4 times daily at 08 : 00, 12 : 00, 16 : 00, and 20 : 00 and had free access to water. The experiment lasted 28 days.

On 29 d, piglets were sacrificed. Digesta from colon was collected to keep in a sterile tube and then frozen at −80°C for the analysis of SCFAs and bacterial community. The colon section was weighed after digesta removed. Mucosal scrapings from the colon were prepared and stored at −80°C to detect gene expression of barrier function.

### 2.3. Microbial DNA Extraction and Sequencing

Total bacterial DNA of colonic digesta was extracted from each sample by using CTAB/SDS method. 1% agarose gels were used for monitoring the concentration and purity of DNA. Following monitoring, DNA was diluted to 1 ng/*μ*l using sterile water according to its concentration. The bacterial 16S rRNA gene amplification (V3–V4 fragments) was conducted using the barcoded primer pair 343F/806R set (343F: TACGGRAGGCAGCAG, 806R: GGACTACHVGGGTWTCTAAT). All PCRs (30 *μ*L) were carried out with 15 *μ*L of Phusion® High-Fidelity PCR Master Mix (New England Biolabs), 0.2 *μ*M of forward and reverse primers, and about 10 ng template DNA. Thermal cycling reactions were performed by the following cycle program: initial denaturation at 98°C for 1 min, followed by 30 cycles of denaturation at 98°C for 10 s, annealing at 50°C for 30 s, with a final elongation at 72°C for 5 min. All PCR products were purified using the electrophoresis in agarose gels and SanPrep DNA Gel Extraction Kit (Sangon Biotech, Shanghai, China). Samples with a bright main strip between 400 and 450 bp were chosen for further experiments. All PCR products were mixed in equal density ratios. The library quality was assessed on the Qubit@ 2.0 Fluorometer (Thermo Scientific) and Agilent Bioanalyzer 2100 system. At last, the library was sequenced on an Illumina HiSeq platform. All reads were deposited in the National Center for Biotechnology Information (NCBI) and can be accessed in the Short Read Archive (SRA) under accession number PRJNA493943.

### 2.4. Bioinformatics Analysis

Paired-end reads from the original DNA fragments are merged by using Fast Length Adjustment of SHort reads (FLASH)—a very fast and accurate analysis tool which is designed to merge paired-end reads when there are overlaps between reads 1 and reads 2 [16]. Paired-end reads were assigned to each sample according to the unique barcodes. Chimeric sequences were removed using USEARCH based on the UCHIME algorithm [17]. The microbial diversity was analyzed using Quantitative Insights Into Microbial Ecology (QIIME) software package [18]. Sequences were assigned to the same Operational Taxonomic Unit (OTU) with a 97% similarity threshold. Alpha diversity was determined based on the number of observed species, Shannon index, Simpson index, Chao1, ACE, goods_coverage, and PD_whole_tree. QIIME calculates both weighted and unweighted UniFrac, which are phylogenetic measures of beta diversity. We used weighted UniFrac for principal coordinate analysis (PCoA). PCoA helps to get principal coordinates and visualize them from complex, multidimensional data.

### 2.5. qPCR Analysis of Bacterial Groups

Quantitative detection of the relative abundance of Bacteroidetes, Firmicutes, *Roseburia*, *Prevotella*, and *Ruminococcus* [19] in all samples was performed by real-time PCR using SYBR Premix Ex Taq reagents (TaKaRa Biotechnology (Dalian), China). The primers (Table 2) and amplification program were followed as the methods mentioned by Bergström et al. [19]. A reaction was run in a volume of 11 *μ*l with 5.5 *μ*l 2 × SYBR Green PCR Master Mix, 0.4 *μ*L of each primer (100 nmol/L), 2.7 *μ*L nuclease-free water, and 2 *μ*L template DNA. The universal bacterial reference primer set was selected for calculating the abundance of target bacterial groups.

### 2.6. Detection of SCFA Concentrations

The SCFA concentrations from colonic digesta were evaluated using gas chromatography. Each sample (1 g) was thawed and suspended in 2 ml distilled water in a screw-capped tube. After 30 min at 4°C, the tubes were centrifuged at 5,000 × g for 10 min. 1 mL supernatant was transferred to a new sterile tube and mixed with 0.2 mL 25% metaphosphoric acid and 23.3 *μ*L 210 mmol/L crotonic acid. After stood for 30 min, the tubes were centrifuged at 10,000 × g for 10 min, and 300 *μ*L supernatant was transferred to another sterile tube, and then 900 *μ*L methanol was added. After centrifuged at 10,000 × g for 10 min, 100 *μ*L supernatant was transferred to a sterile tube. The SCFAs (acetate, propionate, and butyrate) were quantified in a gas chromatographic system (VARIAN CP-3800, America).

### 2.7. ELISA Analysis of Secretory IgA Concentration

According to a 1 : 9 ratio (g/mL), mucosa from the colon was homogenized using physiological saline. The homogenate was centrifuged at 3,500 × g and 4°C for 10 min. Then, the supernatant was taken for the determination of sIgA using commercially available ELISA kit (Chenglin, Beijing).

### 2.8. RT-qPCR Analysis for Gene Expression

According to the manufacturer's instructions, total RNA from the colonic mucosa was isolated using TRIzol reagent (TaKaRa Biotechnology (Dalian), China). The yield and purity of total mRNA were measured using a spectrophotometer (Beckman Coulter DU 800, Beckman Coulter Inc, Brea, USA), and an optical density 260: 280 (OD260: OD280) ranging from 1.8 to 2.0 was considered a very low degree of contamination [20]. The integrity of RNA was analyzed by agarose gel electrophoresis. The RNA samples were reversely transcribed into complementary DNA using RT Reagents (TaKaRa Biotechnology (Dalian), China) according to the manufacturer's instructions. After reverse transcription, gene expressions of zonula occludens 1 (*ZO-1*), occludin (*OCLN*), claudin 1 (*CLDN-1*), mucin 1 (*MUC1*), mucin 2 (*MUC2*), interleukin-10 (*IL-10*), interleukin-1*β* (*IL-1β*), and tumor necrosis factor-*α* (*TNF-α*) in colon were analyzed by real-time quantitative PCR using SYBR Premix Ex Taq reagents (TaKaRa Biotechnology (Dalian), China) and CFX-96 Real-Time PCR Detection System (Bio-Rad Laboratories, Richmond, CA) as described by Mao [21]. The primers (Table 3) were purchased from Invitrogen (Shanghai, China). Cycling conditions were performed as previously described [21]. Each sample was determined in triplicate on the same PCR plate, and the mean values were used for the statistical analysis. Relative gene expression to the reference gene (*β-actin*) was used for normalization, and the relative mRNA expression levels of the target gene in comparison with the reference gene were calculated by the 2^–ΔΔCT^ method [22].

### 2.9. Statistical Analysis

Each piglet was considered as the statistical unit. All data were analyzed as a randomized complete block design using the MIX of SAS (SAS Inst. Inc., Cary, NC). Differences in the weight index of the colon, the relative abundance of certain bacterial phyla, families, or genera, alpha diversity, the concentrations of SCFAs, and gene expressions among treatments were analyzed with one-way ANOVA if the data were in line with a normal distribution (sig > 0.05). Once the results were not in line with normal distribution, they were analyzed by a nonparametric test of significance. The microbiome field results were analyzed using false discovery rate (FDR) to correction with a *q* < 0.1. The results were presented as means ± SE. *P* < 0.05 was considered a significant difference.

## 3. Results

### 3.1. Sequence Analysis

A total of 1,935,292 raw reads with an average of 80,637 ± 10,162 sequences per sample were generated in 24 samples. After removing the low-quality sequences, 1,372,259 valid sequences with an average length of 418 bp were obtained. A total of 1815 OTUs were obtained, which could be identified to 26 bacterial phyla and 312 bacterial genera.

### 3.2. Microbial Diversity in Different Groups

Alpha diversity, be expressed as observed_species, Shannon's diversity index, Simpson index, Chao1, ACE, goods_coverage, and PD_whole_tree, was calculated in microbial diversity analysis within the community (Table 4). Chao1 and ACE in the SDF group were the highest among all groups (*P* < 0.05). Observed_species was higher in the SDF group than in the IDF and MDF groups (*P* < 0.05).

**Figure 1:**
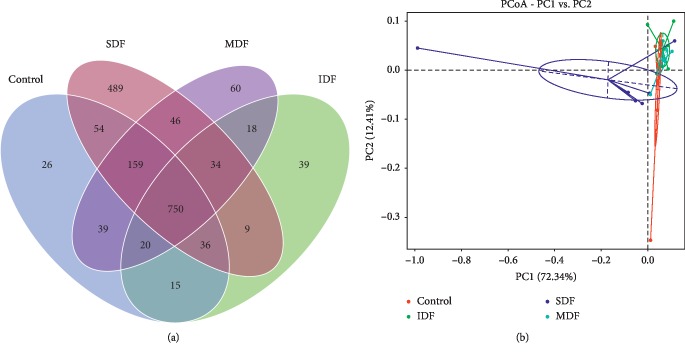
(a) Venn diagram shows the unique and shared OTUs in different groups (*n*=6). (b) Principal coordinate analysis (PCoA) of bacterial community structures in different groups; each represented by one color (*n*=6). PCoA shows distinct bacterial communities for the four different groups. CON, control; IDF, 1% insoluble fiber; SDF, 1% soluble fiber; MDF, 0.5% insoluble fiber +0.5% soluble fiber.

Venn diagram showed the shared and unique OTUs among different groups (Figure 1(a)). There were 1099, 921, 1577, and 1126 OTUs in the CON, IDF, SDF, and MDF groups, respectively. The CON group had 26 unique OTUs, the IDF group had 39 unique OTUs, the SDF group had 489 unique OTUs, and the MDF group had 60 unique OTUs. According to PCoA, the colonic bacterial community structures of the SDF group were obviously separated from other groups (Figure 1(b)).

### 3.3. Composition and Abundance of Bacterial Communities in Different Groups

**Figure 2:**
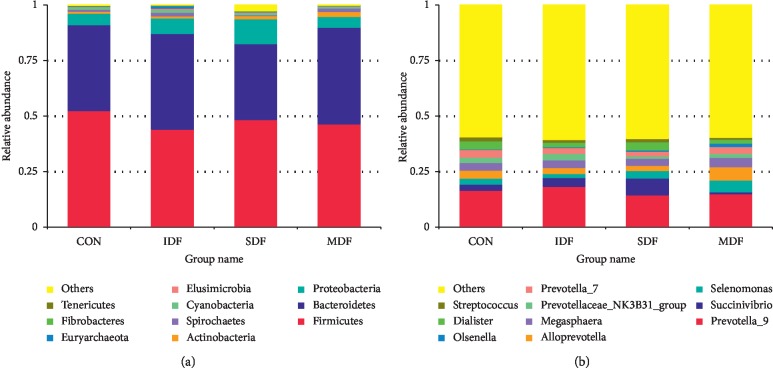
The relative abundances of top 10 phyla (a) and genera (b) in different groups (*n*=6). Each bar represents the average relative abundance of each bacterial taxon within a group. The top 10 most abundant taxa are shown. CON, control; IDF, 1% insoluble fiber; SDF, 1% soluble fiber; MDF, 0.5% insoluble fiber +0.5% soluble fiber.

At the phylum level, a total of 26 phyla were detected in all samples. The most predominant bacterial phyla were Firmicutes and Bacteroidetes, which accounted for 47.9% and 40% of sequences, respectively (Figure 2(a)). The relative abundance of Bacteroidetes in the IDF and MDF groups was significantly higher than that in the SDF group (*P* < 0.05). The relative abundance of Proteobacteria in the SDF group and Euryarchaeota in the IDF group was the highest among all groups, respectively (*P* < 0.05). SDF diet increased the relative abundance of Actinobacteria compared with the CON group (*P* < 0.05).

The most relatively abundant bacterial families were Prevotellaceae and Veillonellaceae. SDF-fed piglets had a lower relative abundance of Prevotellaceae than the CON, IDF, and MDF groups (*P* < 0.05). Piglets from the MDF group had a lower (*P* < 0.05) relative abundance of Lachnospiraceae than those from the CON group and had a higher (*P* < 0.05) relative abundance of Enterobacteriaceae than those from the IDF group (Table 5 and Figure 2(a)).

A total of 312 different genera were detected. The most relatively abundant genera included *Prevotella_9*, *Succinivibrio*, *Selenomonas*, *Alloprevotella*, *Megasphaera*, *Dialister*, *Prevotellaceae_NK3B31_ group*, *Prevotella_7*, *Olsenella*, and *Streptococcus* (Figure 2(b)). The relative abundance of *Dialister*, *Pseudobutyrivibrio, Ruminococcus_2, Ruminiclostridium,* and *Clostridium_sensu_stricto_6* was significantly higher (*P* < 0.05) in the CON group than in the IDF, SDF, and MDF groups. *Phascolarctobacterium, Coprococcus_1, Prevotella_1, Ruminococcaceae_UCG-008, Leeia,* and *Treponema_2* were significantly abundant in the IDF group than in the CON, SDF, and MDF groups (*P* < 0.05). Feeding SDF diets increased the relative abundance of *Blautia, Solobacterium, Syntrophococcus, Olsenella, Atopobium, Succinivibrio,* and *Weissella*(*P* < 0.05) when compared with the CON, IDF, and MDF groups. *Selenomonas, Sharpea, Alloprevotella, unidentified_Veillonellaceae,* and *Phascolarctobacterium* were significantly increased in the MDF group when compared with the CON, IDF, and SDF groups (*P* < 0.05) (Table 6 and Figure 2(b)).

### 3.4. Relative Abundance of Specific Bacteria in Different Groups Using qPCR

As shown in Table 7, the relative abundance of Bacteroidetes in the IDF group was higher than that in the SDF group (*P* < 0.05). The CON group had the highest relative abundance of *Prevotella*(*P* < 0.05).

### 3.5. Short-Chain Fatty Acid Concentrations in Different Groups

**Figure 3:**
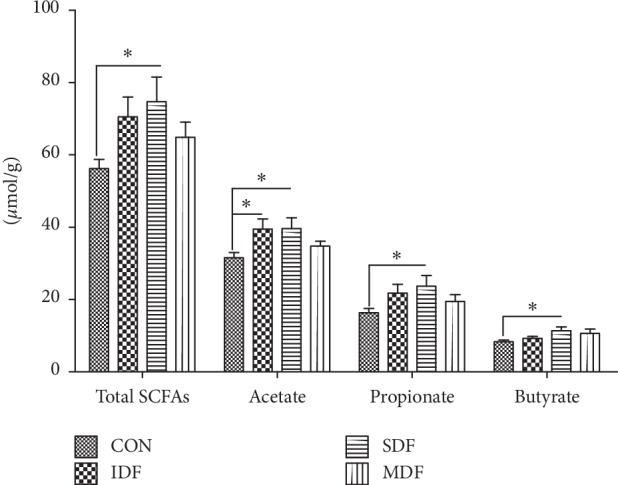
Short-chain fatty acid concentrations in colonic digesta of different groups (*μ*mol/g) (*n*=6). Stars above the bars (*∗*) indicate statistical significance (*P* < 0.05) among the four groups. CON, control; IDF, 1% insoluble fiber; SDF, 1% soluble fiber; MDF, 0.5% insoluble fiber +0.5% soluble fiber.

The SCFA concentrations in the colonic digesta are presented in Figure 3. Compared with the CON group, SDF diet increased the concentrations of total SCFAs, acetate, propionate, and butyrate (*P* < 0.05), while IDF diet increased acetate concentration (*P* < 0.05).

### 3.6. Weight Index and Gene Expressions of Colon in Different Groups

**Figure 4:**
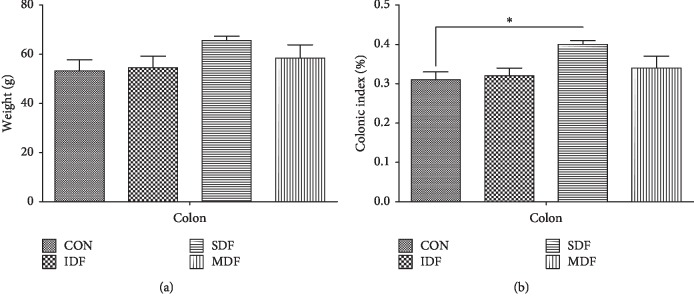
Colonic weight (a) and weight index (b) in different groups (*n*=6). The colonic weight index was calculated by colonic weight index (%) = colonic weight (g)/body weight (g) × 100%. Letters above the bars (a, b) indicate statistical significance (*P* < 0.05) among the four groups. CON, control; IDF, 1% insoluble fiber; SDF, 1% soluble fiber; MDF, 0.5% insoluble fiber +0.5% soluble fiber.

**Figure 5:**
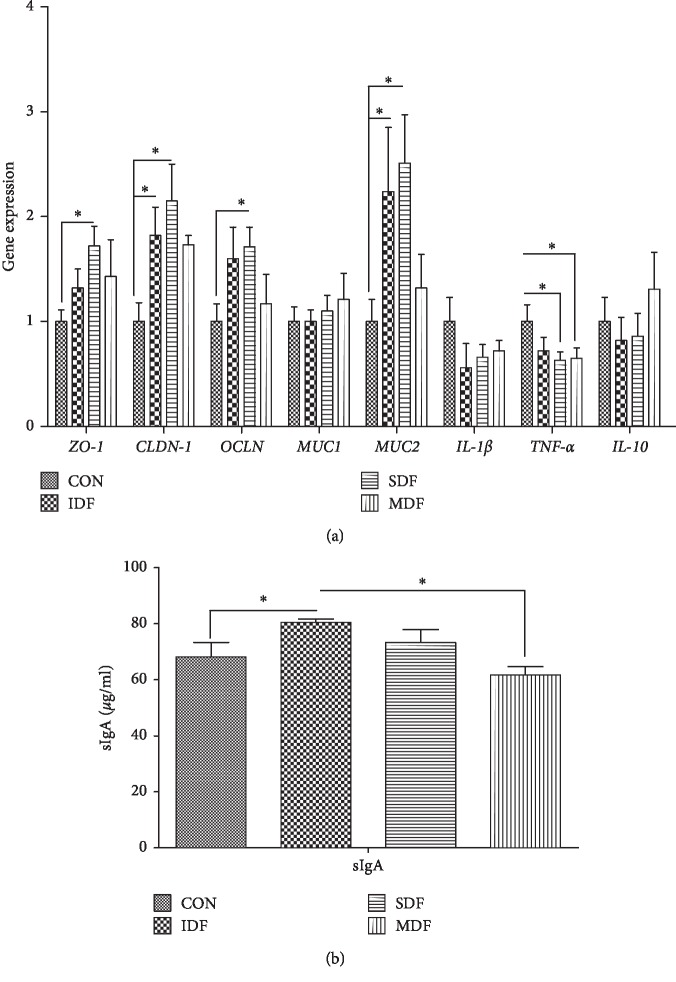
Gene expressions involved in the intestinal barrier (a) and sIgA concentration (b) in different groups (*n*=6). Letters above the bars (a, b) indicate statistical significance (*P* < 0.05) of gene expression among the four groups. CON, control; IDF, 1% insoluble fiber; SDF, 1% soluble fiber; MDF, 0.5% insoluble fiber +0.5% soluble fiber; *ZO-1*: zonula occludens 1; *OCLN*: occludin; *CLDN-1*: claudin 1; *MUC1*: mucin 1; *MUC2*: mucin 2; *TNF-α*: tumor necrosis factor-*α*; sIgA: secretory IgA.

The weight index of colon in the SDF group was higher than that in the CON and IDF groups (*P* < 0.05) (Figure 4). The effects of dietary fibers on colonic gene expressions and sIgA concentration are presented in Figure 5. Compared with the control group, SDF diet increased mRNA expressions of *ZO-1*, *CLDN-1*, *OCLN*, and *MUC2* while decreased mRNA expression of *TNF-α*(*P* < 0.05). An increase in *CLDN-1* and *MUC2* mRNA levels and sIgA concentration was observed in the IDF group when compared with the CON group (*P* < 0.05). Compared with the CON group, MDF diet increased mRNA expression of *CLDN-1* and decreased mRNA expression of *TNF-α*(*P* < 0.05).

## 4. Discussion

Dietary fiber has been shown to be degraded in the hindgut of animals and influenced the abundance and diversity of intestinal microbiota [23, 24]. Changes of fiber components led to a change in the composition of the microbiota [25, 26]. A remarkably higher relative abundance of the phylum Bacteroidetes and Euryarchaeota and the genus *Prevotella*, *Phascolarctobacterium, Ruminococcaceae*, *Coprococcus*, *Leeia*, and *Treponema* was found in the IDF group. SDF diet increased the relative abundance of the phylum Proteobacteria and Actinobacteria and the genus *Blautia*, *Solobacterium*, *Syntrophococcus*, *Weissella*, *Olsenella*, *Atopobium*, and *Succinivibrio*. MDF diet increased the relative abundance of the phylum Bacteroidetes and the genus *Selenomonas*, *Phascolarctobacterium, Sharpea*, and *Alloprevotella*. All these results indicated that different types of fiber could selectively regulate intestinal bacteria. It was reported that a significant increase in Actinobacteria mostly resulted in an increase in *Bifidobacterium* [27]. However, there was no significant difference and we did not find an increase in *Bifidobacterium* although the abundance of Actinobacteria was increased in the SDF group, which may result from the low relative abundance of *Bifidobacterium* in the colon of pigs.

The *α*-diversity index (observed_species, chao1, and ACE) of colonic bacteria was significantly increased in the SDF group when compared to other groups. The results were similar with a study in mice showing an increase in bacterial diversity after *β*-glucan supplementation [28]. These results indicated that the supplementation of soluble fiber but not insoluble fiber may increase the diversity of colonic microbes. Firmicutes, Bacteroidetes, Proteobacteria, and Actinobacteria were the most predominant phyla in all piglets, which were consistent with previous studies in pigs and humans [29–31]. Inulin was reported to increase the abundance of Actinobacteria and decrease Bacteroidetes *in vitro* fermentation, while cellulose increased the abundance of Bacteroidetes and decreased Firmicutes [27]. In the present study, SDF-fed pigs showed a higher abundance of Actinobacteria and a lower abundance of Bacteroidetes; meanwhile, IDF diet increased the abundance of Bacteroidetes and decreased the abundance of Firmicutes. Although the change of Firmicutes was not significant, these results showed that the two fibers have consistent effects on microorganisms *in vitro* and *in vivo.*

In our results, IDF diet increased the concentration of acetate, while SDF diet increased the concentration of total SCFAs (acetate, propionate, and butyrate). The amounts of SCFAs in the colon digesta depended on several factors such as the composition of microbiota and types of fiber. The amounts of acetate and propionate correlate positively with Bacteroidetes and genus *Blautia* within Firmicutes [32, 33]. Higher relative abundances of Bacteroidetes were found in the IDF and MDF groups, while animals from the SDF group showed a higher abundance of *Blautia*. However, a higher concentration of acetate was only found in piglets fed IDF and SDF diets. The reason may be that feeding MDF diet promoted the growth of non-acetate-producing bacteria. SDF-fed piglets had a higher concentration of butyrate and the abundance of Actinobacteria, which confirmed the study that Actinobacteria produced high amounts of colonic butyrate production [34]. Meanwhile, the production of SCFAs was related to the composition of dietary fiber available for bacteria [35]. All saccharide composition of dietary fiber can be utilized for acetate formation and thus increases the acetate concentration [36]; in addition, butyrate can be produced by the fermentation of fructans [37]. The fermentation of soluble carbohydrates leads to a large amount of propionate production [38, 39]. The concentration of SCFAs (acetate, propionate, and butyrate) in the IDF and SDF groups was consistent with previous studies, which suggested that fermentation patterns of dietary fiber were closely related to the diversity of bacterial community and composition of fiber.

The intestinal barrier is consisted of tight junction proteins (ZO-1, CLDN1, and OCLN), the mucus, and immunological components like sIgA [32, 40]. Previous studies showed that dietary fiber proves intestinal barrier function in humans and animals [41]. High fiber diet increased the capacity of mucin secretion in the gastrointestinal tract [42]. MUC2 is the main secretory mucin in colon [43], and thus, IDF and SDF diets upregulated MUC2 mRNA level, suggesting an increased mucin secretion. Arabinoxylan in wheat was reported to increase intestinal sIgA concentrations in weaned piglets, in line with our results in animals fed IDF, which may be ascribed to a low concentration of toxic products [44]. Tight junction proteins (ZO-1, CLDN1, and OCLN) are highly organized structures that maintain an effective barrier against the invasion of harmful substances [45]. In the present study, different fiber groups did not have the same qualitative or quantitative effects on colonic barrier functions, while SDF diet had a better effect than other diets. Furthermore, SDF and MDF diets might promote barrier function by downregulating *TNF-α* gene expression since TNF-α was reported to reduce the tight junction protein expression [46]. As fiber is the main substrate for bacterial fermentation, it might regulate mucosal barrier function by supporting more diversified bacterial communities and increasing concentrations of SCFAs [47, 48]. SDF diet increased the microbial diversity and SCFA concentrations in colon, which might be the reason that SDF diet improved the barrier functions more effectively than other groups.

Research studies have increasingly suggested that probiotic bacteria play an important role in regulating gut barrier integrity [49]. *Coprococcus* was reported to regulate immune responses presumably through the production of IgG [50]. A higher concentration of sIgA found in the IDF group was probably also associated with the increase of *Coprococcus.* Prevotellaceae was increased in colorectal cancer patients while *Blautia* and *Phascolarctobacterium* were reduced [51]. *Atopobium* was considered to be useful for human health since an inverse correlation between its number and inflammatory bowel disease [52]. In the current study, SDF diet significantly increased the alpha diversity of colonic bacteria and the relative abundance of *Blautia* and *Atopobium* and decreased the relative abundance of Prevotellaceae. IDF and MDF diets increased the relative abundance of *Phascolarctobacterium*. Meanwhile, all fiber groups upregulated gene expressions of *ZO-1*, *OCLN*, *CLDN-1*, *MUC1*, and *MUC2* and downregulated gene expressions of *IL-1β* and *TNF-α* in the colon. The results suggested that different fiber supplementation regulated gut barrier function by stimulating the growth of different bacterial species. Probiotic mixture protected the epithelial barrier and increased the OCLN and ZO-1 expression by activating the p38 and ERK signaling pathways, while reversed the effects of TNF-*α* [53], which suggested that dietary fiber might regulate barrier function by p38 and ERK signaling pathways. *L. acidophilus* could activate a pathogen-associated molecular pattern receptor, Toll-like receptor 2 (TLR2) in intestinal epithelial cell lines, and enhance the phosphorylation of NF-*κ*B p65 and p38 mitogen-activated protein kinase (MAPK), which indicated another possible mechanism [54]. However, the underlying mechanism needs to be verified by further studies.

The SCFAs (acetate, propionate, and butyrate) produced as end metabolites by the microbiota were reported to improve gut barrier function [55]. An increase in acetate concentration in the IDF and SDF groups might be the partial reason for the increase of *MUC2* mRNA level, since acetate has been shown to prevent inflammatory bowel diseases by inducing mucin secretion in mucin-deficient mice [56]. Significant positive correlations between colonic propionate concentrations and *TFF* expression were observed in rats [43]. Diet supplementation with butyrate inhibited the disruption of the intestinal epithelial barrier induced by high-fat diet via upregulating the gene expression of *CLDN-1* [57]. In the present study, the highest concentration of SCFAs might be one of the reasons that SDF diet was more efficient in regulating colonic barrier function than other groups.

## 5. Conclusions

In summary, different types of fibers had different effects on the colonic barrier function by selectively modulating bacteria and SCFAs. Insoluble fiber like cellulose increased the relative abundance of Bacteroidetes, Euryarchaeota, *Phascolarctobacterium,* and *Coprococcus*, while soluble fiber like inulin stimulated Actinobacteria, Proteobacteria, *Blautia,* and *Atopobium*. Furthermore, feeding soluble fiber led to a higher concentration of SCFAs, microbial diversity, and community richness than insoluble fiber and then helped to improve the intestinal barrier function.

## Figures and Tables

**Table 1:** Ingredients and nutrient composition of the basal diet (air dry basis).

Item	Content
Ingredient composition (%)	
Maize	30.26
Extruded maize	29.62
Extruded soybean meal	8.45
Soybean meal	9.50
Fish meal	4.00
Whey powder	6.00
Sucrose	2.50
Soybean protein concentrate	6.10
Soybean oil	1.50
DL-Met, 99%	0.07
L-Lys-HCl, 78%	0.26
L-Thr, 98.5%	0.01
L-Trp, 98%	0.01
Choline chloride, 50%	0.15
NaCl	0.20
Limestone	0.61
Monocalcium phosphate	0.41
Vitamin premix^1^	0.05
Trace mineral premix^2^	0.30

Total	100.00
Calculated composition	
Digestible energy (Mcal/kg)	3.52
Crude protein (%)	19.02
Calcium (%)	0.75
Total phosphorus (%)	0.56
Available phosphorus (%)	0.37
Digestible Lys (%)	1.29
Digestible Met (%)	0.39
Digestible Trp (%)	0.22
Digestible Thr (%)	0.78
Digestible Met + Cys (%)	0.61

^1^Vitamin premix provided the following per kg of diets: vitamin A, 9000 IU; vitamin D_3_, 3000 IU; vitamin E, 20.0 IU; vitamin K_3_, 3.0 mg; vitamin B_1_, 1.5 mg; vitamin B_2_, 4.0 mg; vitamin B_6_, 3.0 mg; vitamin B_12_, 0.2 mg; niacin, 30.0 mg; pantothenic acid, 15.0 mg; folic acid, 0.75 mg; biotin, 0.1 mg. ^2^Mineral premix provided the following per kg of diets: 100 mg Fe (as FeSO_4_·H_2_O); 6 mg Cu (as CuSO_4_·5H_2_O); 100 mg Zn (as ZnSO_4_·H_2_O); 4 mg Mn (as MnSO_4_·H_2_O); 0·14 mg I (as KI); 0·3 mg Se (as Na_2_SeO_3_).

**Table 2:** Sequences of primers and probes for intestinal bacteria.

Bacteria	Primer sequence (5′–3′)^1^	*A*_T_^2^ (°C)	Size (bp)
Firmicutes	F: TGAAACTCAAAGGAATTGACG	60	157
	R: ACCATGCACCACCTGTC		

Bacteroidetes	F: GGAACATGTGGTTTAATTCGATGAT	60	126
	R: AGCTGACGACAACCATGCAG		

*Roseburia*	F: TACTGCATTGGAAACTGTCG	60	230
	R: CGGCACCGAAGAGCAAT		

*Prevotella*	F: CACCAAGGCGACGATCA	60	283
	R: GGATAACGCCTGGACCT		

*Ruminococcus*	F: GAGTGAAGTAGAGGTAAGCGGAATTC	60	220
	R: GCCGTACTCCCCAGGTGG		

Universal	F: ACTCCTACGGGAGGCAGCAGT	60	177–179
	R: GTATTACCGCGGCTGCTGGCAC		

^1^F: forward primer; R: reverse primer. ^2^*A*_T_: annealing temperature.

**Table 3:** Primer sequences for RT-PCR amplification.

Gene^1^	Primer sequence (5′–3′)^2^	*A*_T_^3^ (°C)	Size (bp)
*MUC1*	F: GTGCCGCTGCCCACAACCTG	60	141
	R: AGCCGGGTACCCCAGACCCA		

*MUC2*	F: GGTCATGCTGGAGCTGGACAGT	60	181
	R: TGCCTCCTCGGGGTCGTCAC		

*CLDN1*	F: GCCACAGCAAGGTATGGTAAC	60	140
	R: AGTAGGGCACCTCCCAGAAG		

*OCLN*	F: CTACTCGTCCAACGGGAAAG	60	158
	R: ACGCCTCCAAGTTACCACTG		

*ZO-1*	F: CAGCCCCCGTACATGGAGA	60	114
	R: GCGCAGACGGTGTTCATAGTT		

*IL-10*	F: TAATGCCGAAGGCAGAGAGT	57.9	134
	R: GGCCTTGCTCTTGTTTTCAC		

*TNF-α*	F: CGTGAAGCTGAAAGACAACCAG	57.9	121
	R: GATGGTGTGAGTGAGGAAAACG		

*IL-1β*	F: CAGCTGCAAATCTCTCACCA	53	112
	R: TCTTCATCGGCTTCTCCACT		

*β-actin*	F: TCTGGCACCACACCTTCT	60	114
	R: TGATCTGGGTCATCTTCTCAC		

^1^ *MUC1*: mucin 1; *MUC2*: mucin 2; *CLDN-1*: claudin 1; *OCLN*: occludin; *ZO-1*: zonula occludens 1; *TNF-α*: tumor necrosis factor-*α*. ^2^F: forward primer; R: reverse primer. ^3^*A*_T_: annealing temperature.

**Table 4:** Comparison of alpha diversity index in different groups^1^.

Item^2^	CON	IDF	SDF	MDF
Observed_species	629.17 ± 49.21^ab^	619.33 ± 21.98^b^	727.67 ± 45.12^a^	620.5 ± 11.84^b^
Shannon	6.55 ± 0.17	6.55 ± 0.18	6.68 ± 0.07	6.38 ± 0.13
Simpson	0.97 ± 0.00	0.97 ± 0.01	0.97 ± 0.01	0.97 ± 0.00
Chao^1^	671.28 ± 52.31^b^	667.92 ± 23.15^b^	795.28 ± 61.11^a^	664.10 ± 11.54^b^
ACE	670.84 ± 53.12^b^	664.54 ± 22.40^b^	788.28 ± 53.55^a^	667.43 ± 10.82^b^
Goods_coverage	0.9983 ± 0.00	0.9983 ± 0.00	0.9975 ± 0.00	0.998 ± 0.00
PD_whole_tree	39.68 ± 2.77	37.25 ± 1.16	41.94 ± 1.84	39.94 ± 0.74

^a-b^Means within rows with different letters differ significantly (*P* < 0.05). ^1^Values are presented as means ± SE of six replicates per dietary treatment. ^2^CON, control; IDF, 1% insoluble fiber; SDF, 1% soluble fiber; MDF, 0.5% insoluble fiber +0.5% soluble fiber.

**Table 5:** Relative abundance (%) of main bacterial phyla and family in different groups^1^.

Item^2^	CON	IDF	SDF	MDF
*Phyla*				
Firmicutes	52.499 ± 5.86	44.066 ± 3.20	48.426 ± 2.28	46.461 ± 2.02
Bacteroidetes	38.650 ± 4.32^ab^	43.149 ± 1.12^a^	34.181 ± 3.80^b^	43.519 ± 1.82^a^
Proteobacteria	3.750 ± 1.17^b^	3.581 ± 0.79^b^	11.186 ± 2.76^a^	4.888 ± 0.85^b^
Actinobacteria	0.831 ± 0.15^b^	0.943 ± 0.19^ab^	1.460 ± 0.21^a^	0.932 ± 0.24^ab^
Euryarchaeota	0.114 ± 0.04^b^	1.125 ± 0.44^a^	0.183 ± 0.13^b^	0.086 ± 0.05^b^

*Family*				
Prevotellaceae	36.045 ± 0.78^a^	35.643 ± 1.19^a^	27.153 ± 2.37^b^	34.391 ± 2.08^a^
Veillonellaceae	16.081 ± 2.18	12.450 ± 1.73	15.813 ± 1.04	18.257 ± 3.23
Ruminococcaceae	13.556 ± 2.31	11.127 ± 1.19	12.245 ± 0.72	10.512 ± 1.65
Lachnospiraceae	13.544 ± 2.53^a^	11.020 ± 1.59^ab^	10.731 ± 1.23^ab^	8.294 ± 0.94^b^
Enterobacteriaceae	0.319 ± 0.12^ab^	0.160 ± 0.05^b^	0.433 ± 0.14^ab^	1.276 ± 0.59^a^

^a,b^Means within rows with different letters differ significantly (*P* < 0.05). ^1^Values are presented as means ± SE of six replicates per dietary treatment. ^2^CON, control; IDF, 1% insoluble fiber; SDF, 1% soluble fiber; MDF, 0.5% insoluble fiber +0.5% soluble fiber.

**Table 6:** Relative abundance (%) of main bacterial genera in different groups^1^.

Item^2^	CON	IDF	SDF	MDF
*Selenomonas*	2.361 ± 0.81^b^	1.780 ± 0.55^b^	3.379 ± 0.62^ab^	5.352 ± 1.53^a^
*Megasphaera*	3.637 ± 0.39	3.499 ± 0.40	3.200 ± 0.72	4.218 ± 1.47
*Dialister*	4.615 ± 1.27^a^	2.048 ± 0.56^b^	3.649 ± 0.63^ab^	1.793 ± 0.31^b^
*Pseudobutyrivibrio*	5.010 ± 1.12^a^	4.116 ± 0.96^ab^	3.806 ± 0.62^ab^	2.804 ± 0.35^b^
*Asteroleplasma*	0.852 ± 0.35	0.488 ± 0.23	0.563 ± 0.14	0.616 ± 0.28
*Streptococcus*	0.416 ± 0.10	1.198 ± 0.31	1.457 ± 0.74	0.808 ± 0.24
*Ruminococcaceae_UCG-002*	1.864 ± 0.30	1.669 ± 0.51	2.138 ± 0.31	2.468 ± 0.87
*Mitsuokella*	1.922 ± 0.62	1.091 ± 0.33	1.539 ± 0.32	2.419 ± 0.87
*Eubacterium_ruminantium_group*	1.521 ± 0.97	0.381 ± 0.12	0.179 ± 0.04	0.098 ± 0.04
*Terrisporobacter*	0.872 ± 0.19	1.328 ± 0.43	0.919 ± 0.22	1.115 ± 0.33
*Anaerovibrio*	1.036 ± 0.31	1.815 ± 0.55	0.858 ± 0.27	1.404 ± 0.38
*Eubacterium_coprostanoligenes_group*	1.418 ± 0.47	1.139 ± 0.32	0.957 ± 0.10	0.902 ± 0.24
*Ruminococcaceae_NK4A214_group*	1.296 ± 0.47	0.797 ± 0.12	0.801 ± 0.03	0.768 ± 0.13
*Lachnospiraceae_UCG-005*	0.877 ± 0.12	0.799 ± 0.19	0.728 ± 0.20	0.566 ± 0.20
*Faecalibacterium*	1.817 ± 0.29	1.165 ± 0.15	1.442 ± 0.36	1.039 ± 0.29
*Phascolarctobacterium*	0.242 ± 0.06^b^	0.536 ± 0.10^a^	0.317 ± 0.07^ab^	0.547 ± 0.10^a^
*Ruminococcus_1*	0.661 ± 0.07	0.830 ± 0.15	0.870 ± 0.29	0.507 ± 0.07
*Ruminococcaceae_UCG-005*	0.896 ± 0.26	0.779 ± 0.10	0.845 ± 0.06	0.688 ± 0.11
*Catenibacterium*	0.583 ± 0.13	0.312 ± 0.05	0.520 ± 0.19	0.285 ± 0.13
*Sarcina*	0.579 ± 0.13	0.607 ± 0.06	0.739 ± 0.16	0.460 ± 0.09
*Lactobacillus*	0.508 ± 0.21	0.708 ± 0.10	0.806 ± 0.28	0.642 ± 0.24
*Acidaminococcus*	0.424 ± 0.14	0.319 ± 0.16	0.355 ± 0.14	0.625 ± 0.28
*Blautia*	0.979 ± 0.09^ab^	0.994 ± 0.12^ab^	1.022 ± 0.10^a^	0.691 ± 0.09^b^
*Ruminococcus_2*	0.576 ± 0.27^a^	0.279 ± 0.08^ab^	0.181 ± 0.04^ab^	0.117 ± 0.01^b^
*Subdoligranulum*	0.712 ± 0.06	0.690 ± 0.10	0.908 ± 0.08	0.825 ± 0.10
*Ruminococcaceae_UCG-010*	0.462 ± 0.10	0.445 ± 0.11	0.591 ± 0.16	0.550 ± 0.17
*Ruminococcaceae_UCG-014*	0.698 ± 0.12	0.651 ± 0.16	0.645 ± 0.15	0.520 ± 0.12
*Holdemanella*	0.491 ± 0.08	0.370 ± 0.09	0.543 ± 0.14	0.288 ± 0.13
*Anaerotruncus*	0.519 ± 0.06	0.413 ± 0.06	0.508 ± 0.04	0.418 ± 0.09
*Ruminococcaceae_UCG-008*	0.232 ± 0.04^ab^	0.334 ± 0.11^a^	0.182 ± 0.02^ab^	0.107 ± 0.01^b^
*unidentified_Veillonellaceae*	0.156 ± 0.07^b^	0.048 ± 0.00^b^	0.085 ± 0.02^b^	0.346 ± 0.09^a^
*Sharpea*	0.027 ± 0.01^b^	0.065 ± 0.04^ab^	0.130 ± 0.08^ab^	0.279 ± 0.12^a^
*Solobacterium*	0.188 ± 0.05^ab^	0.105 ± 0.01^b^	0.250 ± 0.04^a^	0.199 ± 0.03^ab^
*Syntrophococcus*	0.061 ± 0.01^ab^	0.030 ± 0.01^b^	0.136 ± 0.05^a^	0.054 ± 0.03^ab^
*Ruminiclostridium*	0.191 ± 0.03^a^	0.155 ± 0.03^ab^	0.139 ± 0.03^ab^	0.108 ± 0.01^b^
*Coprococcus_1*	0.041 ± 0.00^b^	0.079 ± 0.02^a^	0.052 ± 0.01^ab^	0.047 ± 0.01^ab^
*Clostridium_sensu_stricto_6*	0.055 ± 0.02^a^	0.028 ± 0.01^ab^	0.030 ± 0.01^ab^	0.015 ± 0.00^b^
*Weissella*	0.005 ± 0.00^b^	0.011 ± 0.00^b^	0.025 ± 0.00^a^	0.011 ± 0.01^b^
*Prevotella_9*	14.025 ± 2.63	18.249 ± 2.05	14.448 ± 1.45	14.976 ± 2.47
*Alloprevotella*	3.041 ± 0.89^b^	2.705 ± 0.22^b^	2.319 ± 0.11^b^	5.989 ± 1.68^a^
*Prevotellaceae_NK3B31_group*	2.369 ± 0.69	3.020 ± 1.30	1.261 ± 0.29	2.056 ± 0.78
*Prevotella_7*	2.976 ± 1.10	2.669 ± 0.86	2.051 ± 0.69	3.044 ± 1.16
*Prevotella_2*	2.919 ± 0.69	3.424 ± 0.31	2.306 ± 0.34	2.402 ± 0.70
*Prevotella_1*	1.052 ± 0.28^ab^	1.406 ± 0.08^a^	0.918 ± 0.36^ab^	0.528 ± 0.23^b^
*Rikenellaceae_RC9_gut_group*	1.108 ± 0.22	1.351 ± 0.31	0.971 ± 0.09	1.281 ± 0.33
*Prevotellaceae_UCG-003*	0.665 ± 0.25	0.643 ± 0.07	0.728 ± 0.22	0.355 ± 0.06
*Olsenella*	0.044 ± 0.02^b^	0.104 ± 0.08^ab^	0.304 ± 0.12^a^	0.045 ± 0.02^b^
*Atopobium*	0.010 ± 0.01^b^	0.024 ± 0.01^b^	0.104 ± 0.03^a^	0.043 ± 0.03^ab^
*Succinivibrio*	1.038 ± 0.50^b^	0.933 ± 0.46^b^	7.599 ± 2.63^a^	0.885 ± 0.31^b^
*Campylobacter*	0.328 ± 0.10	0.287 ± 0.05	1.242 ± 0.76	0.754 ± 0.27
*Leeia*	0.194 ± 0.11^b^	0.774 ± 0.36^a^	0.110 ± 0.05^b^	0.113 ± 0.06^b^
*Desulfovibrio*	0.508 ± 0.07	0.496 ± 0.07	0.437 ± 0.04	0.445 ± 0.10
*Treponema_2*	0.332 ± 0.10^b^	0.892 ± 0.28^a^	0.318 ± 0.10^b^	0.518 ± 0.16^ab^
*Methanobrevibacter*	0.253 ± 0.01	0.269 ± 0.16	0.071 ± 0.06	0.018 ± 0.007
*Candidatus_Methanoplasma*	0.043 ± 0.02^ab^	0.226 ± 0.13^a^	0.012 ± 0.01^b^	0.026 ± 0.02^ab^

^a,b^Means within rows with different letters differ significantly (*P* < 0.05). ^1^Values are presented as means ± SE of six replicates per dietary treatment. ^2^CON, control; IDF, 1% insoluble fiber; SDF, 1% soluble fiber; MDF, 0.5% insoluble fiber +0.5% soluble fiber.

**Table 7:** The relative abundance (%) of certain bacterial groups in the colonic digesta of different groups^1^.

Item^2^	CON	IDF	SDF	MDF
Firmicutes	21.75 ± 3.15	21.83 ± 2.08	25.02 ± 4.76	22.36 ± 3.82
Bacteroidetes	48.49 ± 4.07^ab^	53.36 ± 4.73^a^	40.57 ± 3.87^b^	41.13 ± 7.96^ab^
*Roseburia*	0.95 ± 0.28	1.31 ± 0.63	1.30 ± 0.38	0.82 ± 0.32
*Prevotella*	33.62 ± 3.69^a^	25.31 ± 1.54^b^	23.34 ± 1.80^b^	20.93 ± 1.98^b^
*Ruminococcus*	3.89 ± 1.17	3.94 ± 0.68	4.72 ± 1.58	4.72 ± 0.68

^a,b^Means within rows with different letters differ significantly (*P* < 0.05). ^1^Values are presented as means ± SE of six replicates per dietary treatment. ^2^CON, control; IDF, 1% insoluble fiber; SDF, 1% soluble fiber; MDF, 0.5% insoluble fiber +0.5% soluble fiber.

## Data Availability

The data used to support the findings of this study are included within the article.
